# Comparative effects of conventional and electronic cigarettes on discoloration and surface roughness of gingiva‐colored dental materials

**DOI:** 10.1111/jopr.14054

**Published:** 2025-04-02

**Authors:** Melek Almila Erdogan, Ülkü Tuğba Kalyoncuoğlu, Bengi Yilmaz Erdemli

**Affiliations:** ^1^ Department of Prosthodontics Faculty of Gulhane Dentistry University of Health Sciences Turkey Ankara Turkey; ^2^ Department of Biomaterials Hamidiye Institute of Health Sciences University of Health Sciences Turkey Istanbul Turkey; ^3^ Gulhane Medical Design and Manufacturing Center (METUM) University of Health Sciences Turkey Ankara Turkey

**Keywords:** cigarette smoking, composite resins, gingival porcelains

## Abstract

**Purpose:**

To evaluate the color changes and surface roughness of two different gingiva‐colored prosthetic materials after exposure to conventional and electronic cigarettes.

**Materials and Methods:**

A total of 60 disk shaped gingiva‐colored porcelain (GC Initial MC Gum, GC Europe NV, Leuven, Belgium) and indirect composite resin (Gradia Plus Gum Shades Heavy Body, GC Europe NV, Leuven, Belgium) specimens, each measuring 2 × 10 mm, were prepared. The porcelain and indirect composite resin samples were divided into two subgroups (*n* = 30). The initial color and roughness values of the samples were measured. A special setup was created to simulate cigarette smoking, where the samples were placed inside and exposed to two different types of cigarette smoke. After exposure, the final color and roughness values of the samples were measured again.

**Results:**

After conventional cigarette exposure, porcelain specimens showed color changes of ΔE*ab = 7.404, ΔE00 = 7.502, and indirect composite resin specimens ΔE*_ab_ = 9.708, ΔE_00_ = 19.501, with significant surface roughness increases in both materials (*p* < 0.05). After electronic cigarette exposure, porcelain specimens had color changes of ΔE*ab = 1.390, ΔE00 = 1.317, and indirect composite resin samples ΔE*ab = 2.523, ΔE00 *=* 2.454. Surface roughness decreased significantly for porcelain (*p* < 0.05) but increased for composite resin which was not statistically significant (*p* > 0.05).

**Conclusion:**

Conventional cigarette exposure resulted in more significant color changes and surface roughness increases in gingiva‐colored materials compared to electronic cigarettes. Indirect composite resins demonstrated lower color stability than porcelains. Patients with gingiva‐colored porcelain and composite resin restorations should be informed about color and surface roughness changes due to smoking.

According to the World Health Organization, tobacco and nicotine addiction is the biggest health threat affecting 1.3 billion people who use tobacco and tobacco products. Conventional cigarette smoking is the most common method for tobacco consumption globally. However, in recent years, many smokers have been replacing this method with “electronic nicotine delivery system (ENDS),” commonly known as electronic cigarettes.^1^, ^2^, ^3^

Cigarette smoking is closely associated with periodontal diseases, which can lead to tooth or bone loss. Ensuring gingival aesthetics plays an important role in the success of prosthetic treatments, particularly in cases of gingival tissue loss due to bone resorption.^4^ Various surgical techniques can apply to the management of gingival aesthetics. However, surgical treatments are not always possible in some cases, such as alveolar resorption, resection, or asymmetry. To address these situations, gingiva‐colored materials are commonly used for reconstruction of lost gingival tissues.^5^ Gingival porcelain and indirect composites are widely preferred materials for veneering frameworks like Cr‐Co, titanium alloys, zirconia, or PEEK.^6^

Porcelain materials are extensively utilized in dentistry due to their biocompatibility, superior optical properties, and stability.^5^, ^7^ However, indirect composites have been developed as an alternative material to overcome certain drawbacks of porcelain materials such as chipping, extra laboratory steps, shrinkage during multiple firings, challenges in repair, chipping, and inability to perform chairside application.^6^, ^7^ On the other hand, indirect composites have limitations in color stability or stable surface roughness because they are not chemically inert materials.^7^, ^8^

Color stability is an important parameter for enhancing aesthetics of restorations.^9^ Color changes can result from multiple factors including the accumulation of colorant agents on rough surfaces, adsorption due to surface degradation, and physical or chemical reactions.^5^, ^10^, ^11^ A previous study has reported that the surface roughness must be below 0.2 µm in order to prevent plaque accumulation and this value is accepted as a critical threshold for clinical applications.^8^, ^12^ For this reason, glazes on porcelain surfaces and surface sealant agents on the indirect composite surfaces are used to achieve smoothness.^13^, ^14^, ^15^ Colorant agents such as tea, coffee, wine, and cigarettes are widely studied in the literature.^10^, ^16^, ^17^ In studies involving cigarettes, conventional cigarettes are the most commonly used type and many of these studies investigated the correlation between surface roughness and color stability of dental materials.^10^, ^17^, ^18^, ^19^, ^20^ On the other hand, recent studies have focused on the effects of electronic cigarettes on the discoloration of dental materials. Various customized methods are used in studies to evaluate the effects of conventional or electronic cigarettes, and these methods are not standardized.^18^, ^21^ To ensure standardization in cigarette studies, the Cooperation Centre for Scientific Research Relative to Tobacco (CORESTA) has recommended method No. 22 for conventional cigarettes and method No. 81 for electronic cigarettes.^22^, ^23^, ^24^

Due to limited literature,^3^, ^21^, ^25^, ^26^ it is challenging to fully evaluate the effects of electronic cigarettes on the surface properties of dental materials. Despite the application of prosthetic treatments for smoking‐induced tooth and surrounding tissue loss, patients regrettably continue using tobacco products. Studies evaluating gingiva‐colored porcelain and indirect composite resins together are limited, and the effects of cigarette products on gingiva‐colored restorations are not well known. Therefore, the present study is focused on the effects of conventional and electronic cigarettes on the discoloration and surface roughness of gingiva‐colored porcelain and indirect composite resin. The null hypotheses were that conventional and electronic cigarettes would not affect the (1) discoloration and (2) surface roughness of gingiva‐colored porcelain and indirect composite resin.

## MATERIALS AND METHODS

**FIGURE 1:**
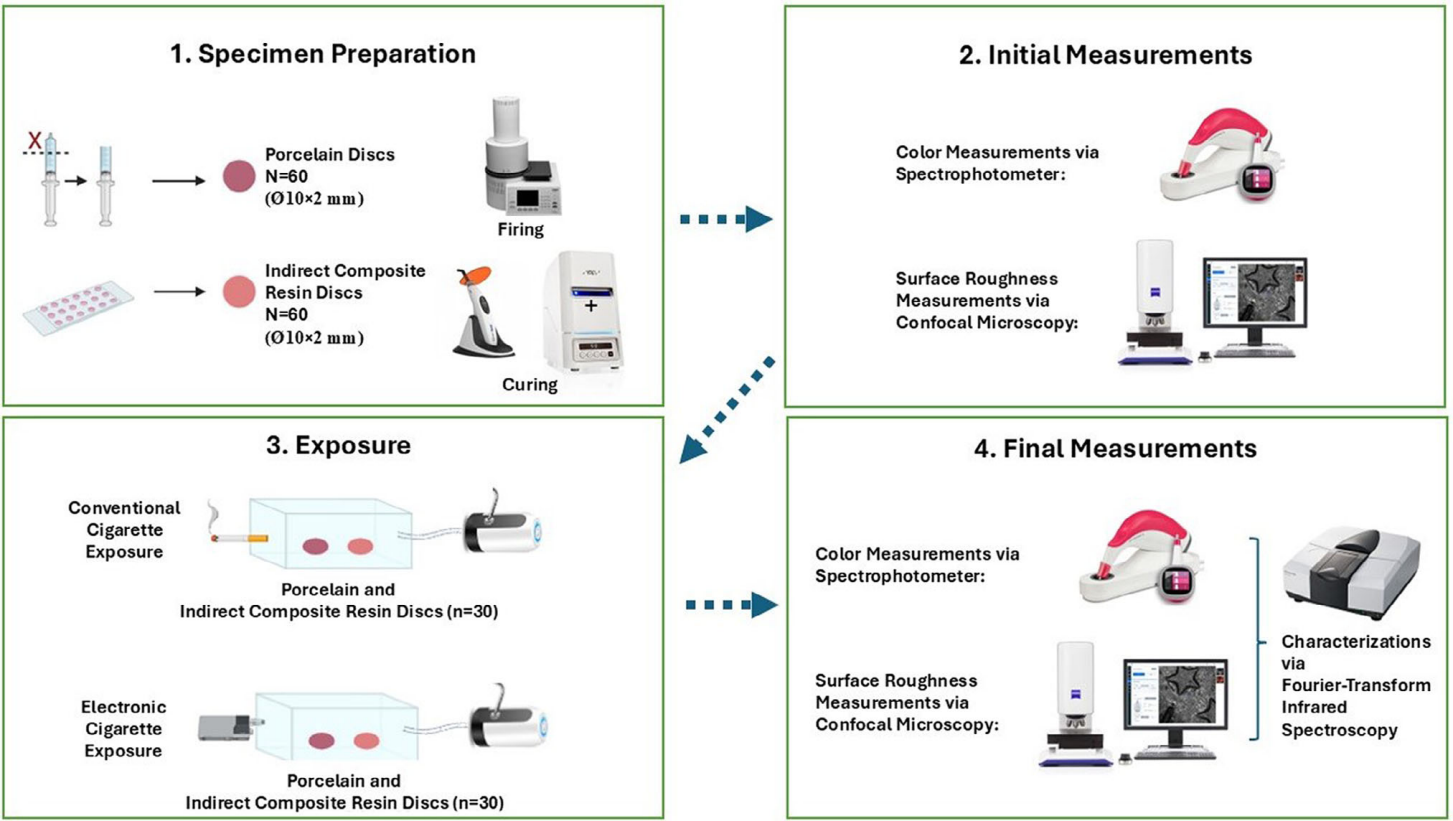
Flowchart illustrating the methodology of the study.

The flowchart of the study is given in Figure 1. A total of 120 disc‐shaped specimens (Ø10 × 2 mm) were prepared from two different gingiva‐colored materials (Porcelain: GC Initial MC Gum, GC Europe NV, Leuven, Belgium; Indirect Composite Resin: Gradia Plus Gum Shades Heavy Body, GC Europe NV, Leuven, Belgium). The brands and characteristics of the materials examined are shown in Table 1.

**TABLE 1:** Materials used in this study.

Materials	Products and brands	Composition	Lot number
Porcelain	GC Initial MC Gum—GU (GC Europe NV Leuven, Belgium)	Feldspathic gingival porcelain	2304141
Indirect composite resin	Gradia Plus Gum Shades Heavy Body‐ GHB‐2 (GC Europe NV Leuven, Belgium)	Resin: UDMA, NGDMA, TMPTMA Filler: Trimodal (pre‐polymerized particles, AlBSiO_4_, SiO_2_, 75 wt%)	230501B
Glaze porcelain	Initial Spectrum Glaze—SPS GL Fluo (GC Europe NV Leuven, Belgium)	Powder and Liquid	Powder: 2307071 Liquid: 2303191
Surface sealant agent	Optiglaze Color—Clear (GC Europe NV Leuven, Belgium)	Light‐cured nano‐filled glaze Methyl methacrylate CAS: 80‐62‐6 (30‐40%), Silica filler (10%), Multifunctional acrylate (50‐60%), diphenyl(2,4,6‐trimethylbenzoyl)‐phosphine oxide (_3‐*<* 5%), Photo initiator (*<* 1%)	2303201

*Note*. The contents of the porcelain material are not disclosed due to brand confidentiality.

Abbreviations: NGDMA, Neopentyl Glycol Dimethacrylate; TMPTMA, Trimethylolpropane Trimethacrylate; UDMA, Urethane Dimethacrylate.

The porcelain specimens (*n* = 60) were fabricated using a mold created from a 10 mL disposable plastic syringe. The needle extension part of the syringe was removed, and the inner rubber piston was coated with composite material to form a flat surface. A 2 mm thickness mark was made on the syringe. The porcelain was prepared and condensed into the syringe mold.^27^ The piston inside the syringe was pushed upward to place the porcelain model on a refractory mat without causing any damage. The prepared porcelain specimens were fired using a heat‐pressing furnace (EP600 Combi, Ivoclar Vivadent AG, Liechtenstein) following the manufacturer's instructions.

All porcelain discs were trimmed using a rotary motor (Stein Weber, Italy) at 15,000 rpm with a 30 µm diamond bur (Frank Dental GmbH, Gmund, Germany). Subsequently, the specimens were sanded with silicon carbide abrasive papers (320–1200 grit; Atlas, Saint‐Gobain Abrasives, Istanbul, Turkey) and then cleaned in an ultrasonic cleaner with distilled water for 10 min, followed by air drying. A glaze of porcelain was applied to all porcelain specimens. Finally, the specimens were stored in distilled water.

To ensure dimensional standardization of the indirect composite resin specimens (Gradia Plus Gum Shades Heavy Body, GC Europe NV, Leuven, Belgium), a plastic mold with 10 mm diameter holes and a thickness of 2 mm was prepared (*n* = 60) and the indirect composite resin was applied into the cavities.

Both surfaces of the indirect composite resin specimens were pre‐polymerized for 40 s using an LED light‐curing device (DTE LUX‐E Plus, Guilin Woodpecker Medical Instrument, Guilin, Guangxi, China; 1200 mW/cm^2^) according to the manufacturer's instructions. The specimens were then placed in a Labolight DUO polymerization unit (GC Europe NV, Leuven, Belgium) and further polymerized for 3 min.

After completing the polymerization process, the specimens were sanded with silicon carbide abrasive papers (320–1200 grit; Atlas, Saint‐Gobain Abrasives, Istanbul, Turkey). Subsequently, polishing was performed using diamond‐impregnated polishing rubbers (Diacomp Plus Twist Set RA 342, EVE Technik, Pforzheim, Germany) at 10,000 rpm for 20 s. Finally, the specimens were cleaned in an ultrasonic cleaner with distilled water for 10 min and air‐dried.

The cleaned surfaces of the indirect composite resin specimens were coated with a surface coating agent (Optiglaze Color Clear, GC Europe NV, Leuven, Belgium) using a clean brush, following the manufacturer's instructions. The specimens were then polymerized in a Labolight DUO polymerization unit (GC Europe NV, Leuven, Belgium) for 90 s.

The initial color measurements of all gingiva‐colored specimens were performed using a spectrophotometer (Easyshade V, VITA Zahnfabrik, Bad Sackingen, Germany).^10^, ^25^ The specimens were placed on a white background inside a measurement box lined with neutral gray fabric and illuminated by a 5500 K fluorescent lamp to simulate daylight. The spectrophotometer was calibrated before use and after every 20 readings. Each specimen was measured three times, and the L, a, b, c, and H values obtained from the measurements were recorded as arithmetic means for use in the CIELAB and CIEDE2000 formulas.^6^

The initial surface roughness measurements of specimens were performed using a widefield confocal microscope (Zeiss Smartproof 5, Carl Zeiss, Jena, Germany). For each specimen, a total area of 500 µm × 500 µm was scanned in fast mode (4 µm) at 20x magnification (C Epiplan‐Apochromat 20x/0.7 DIC, Carl Zeiss, Jena, Germany) from the center of the specimen in 3D imaging mode, with three randomized readings taken without filtering. The obtained images were transferred to automated analysis software (ConfoMap ST 7.4.8076, Carl Zeiss, Jena, Germany). The roughness parameters Rq, Rz, and Ra were determined according to ISO 21920 standards, while Sq, Sz, and Sa values were obtained following ISO 25178 standards by confocal microscope. Among these, Ra which indicates the arithmetical mean roughness and its three‐dimensional equivalent Sa parameters were recorded for further use. This is because Ra is widely used in dentistry as a key parameter for evaluating surface roughness. Additionally, some studies suggest that the surface roughness parameter Sa may also be suitable for assessments conducted using microscopic analysis.^28^

To simulate in vitro smoking, a rigid plastic box^25^, ^29^ with dimensions of 15 cm × 20 cm × 5 cm and an airtight lid was modified by creating holes at the midpoint of two opposite vertical walls to form a smoke passage from the cigarette to vacuum device (BMVA Elektronik, Istanbul, Turkey) equipped with a start‐stop function.

A total of 30 porcelain and 30 indirect composite resin discs were placed in the smoke chamber at a distance of 1.5 cm from the walls, ensuring even distribution.^25^ A transparent silicone adhesive was used to stabilize the specimens.

For conventional cigarette exposure, 20 cigarettes (equivalent to one pack) (Marlboro, Philip Morris, USA) were used.^3^, ^10^, ^17^, ^30^ Each cigarette was assumed to produce 10 puffs, with each puff lasting an average of 2 s and a 60s interval between puffs, following the CORESTA Method No. 22 recommendations.^23^, ^24^ The experiment concluded after the use of 20 cigarettes (200 puffs in total).^3^, ^10^, ^17^

For electronic cigarette exposure, the same setup was used. A disposable electronic cigarette device (5 mg/mL nicotine, Elfbar BC5000 Sweet Menthol, Guangdong Qisitech Co., China) was fully charged and filled, then kept at room temperature.^25^, ^30^ Using the CORESTA Method No. 81 as a reference, exposure was completed with 200 puffs (equivalent to 20 cigarettes), with each puff lasting an average of 3 s and a 30s interval between puffs.^22^, ^25^, ^30^

Following exposure to conventional and electronic cigarette exposures, all porcelain and indirect composite resin specimens were rinsed with distilled water for 1 min. Final measurements were then performed to evaluate color and surface roughness changes, following the same procedure as the initial measurements.

In order to identify the chemical composition and detect potential changes in the functional groups of the specimens, the infrared spectra of the samples were acquired using a Fourier Transform Infrared Spectrometer (FTIR‐ATR, IRTracer‐100, Shimadzu, Japan) equipped with an integrated Attenuated Total Reflection (ATR) accessory. The measurements were performed over a wavenumber range of 400 to 4000 cm^−1^, with a resolution of 4 cm^−1^ and 32 scans per sample.

Taking into account a previous study,^31^ and the parameters of 95% confidence interval, 0.95 power and 0.79 effect size, 30 specimens per group were calculated with the G*Power package (version 3.1, Heinrich‐Heine Dusseldorf University, Dusseldorf, Germany).

Shapiro‐Wilk test was performed to evaluate the normality of the results. Since the measurements followed a normal distribution, parametric tests were applied. Comparisons of independent groups in terms of normally distributed quantitative variables were analyzed by independent *t*‐test. The paired *t*‐test was employed for comparing the before‐after color and surface roughness measurements of the specimens. A statistical analysis software program (IBM SPSS Statistics, v23; IBM Corp) was used for all analyses (*p* < 0.05).

## RESULTS

**FIGURE 2:**
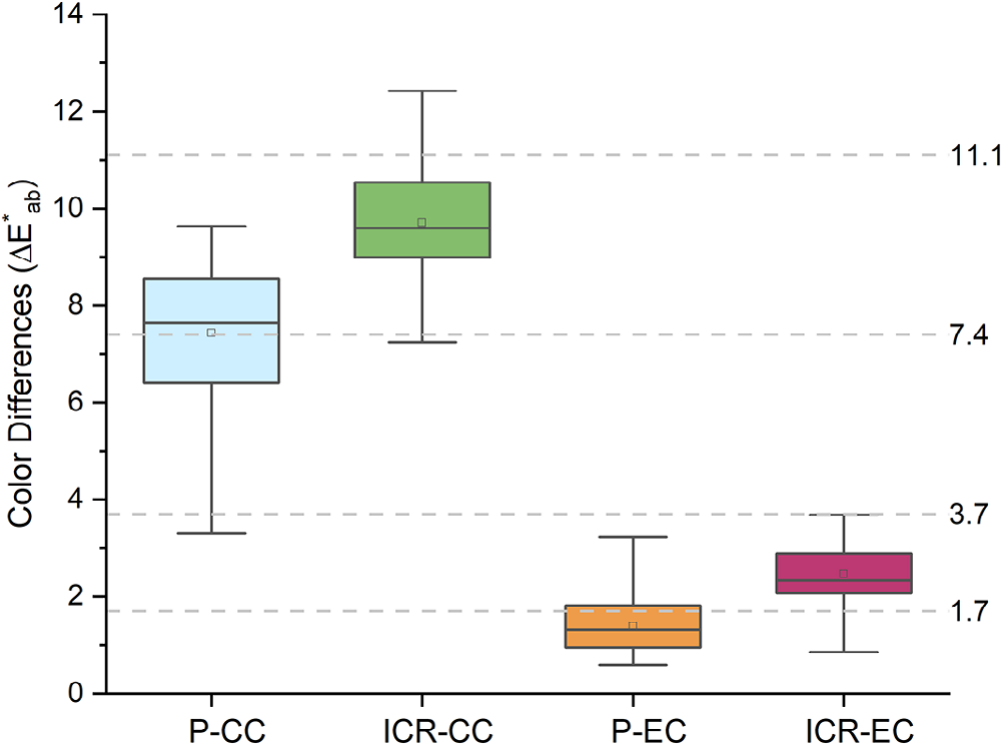
Box plot represents the color differences (△E*ab) of the gingiva‐colored porcelains and indirect composite resins before and after exposure to conventional cigarettes (CC) and electronic cigarettes (EC). P: Porcelain, ICR: Indirect Composite Resin. Interpretation of color differences: excellent match △E*ab≤ 1.7; acceptable match 1.7<△E*ab ≤3.7; moderately unacceptable 3.7<△E*ab≤7.4; clearly unacceptable 7.4<△E*ab≤11.1, and extremely unacceptable △E*ab>11.1.

**FIGURE 3:**
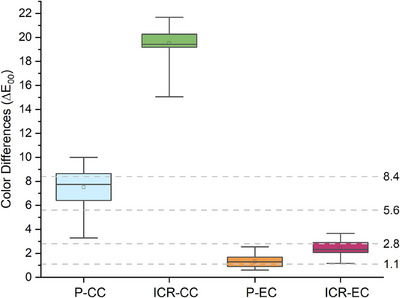
Box plot represents the color differences (ΔE_00_ values) of the gingiva‐colored specimens before and after exposure to conventional cigarettes (CC) and electronic cigarettes (EC). P: Porcelain, ICR: Indirect Composite Resin. Interpretation of color differences: excellent match △E00≤ 1.1; acceptable match 1.1<△E00≤2.8; moderately unacceptable 2.8<△E00≤5.6; clearly unacceptable 5.6<△E00≤8.4, and extremely unacceptable△E00>8.4.

The descriptive and comparative statistics of the color values are shown in Table 2. After conventional cigarette exposure, the L* and a* values of the porcelain and indirect composite resins significantly decreased, while the b* values increased (*p* < 0.05). The ΔE*_ab_ was found to be 7.404 for porcelain specimens and 9.708 for indirect composite resins. The ΔE_00_ was 7.502 for porcelain specimens and 19.501 for indirect composite resins. After electronic cigarette exposure, the L* and a* values of all specimens increased, while the b* value decreased (*p* < 0.05). The ΔE*_ab_ was 1.39 for porcelain specimens and 2.523 for indirect composite resin specimens. The ΔE_00_ values were 1.317 for porcelain specimens and 2.454 for indirect composite resin specimens (Figure 2 and Figure 3).

**TABLE 2:** The descriptive and comparative statistics of the color values.

	Conventional cigarette					Electronic cigarette				
	Gingiva‐colored porcelain		Gingiva‐ colored indirect composite resin		*p value	Gingiva‐ colored porcelain		Gingiva‐ colored indirect composite resin		*p value
	Mean	SD	Mean	SD		Mean	SD	Mean	SD	
**L**_before_	46.056	0.599	58.838	0.648	***0.001***	44.978	0.703	58.531	0.699	***0.001***
**L**_after_	42.713	0.979	54.424	0.745	***0.001***	45.732	0.385	59.927	0.785	***0.001***
**p value	***0.001***		***0.001***			***0.001***		***0.001***		
**a**_before_	30.848	0.869	24.656	0.328	***0.001***	31.633	0.427	24.470	0.290	***0.001***
**a**_after_	30.436	0.687	23.377	0.287	***0.001***	31.800	0.438	24.569	0.316	***0.001***
**p value	***0.007***		***0.001***			***0.021***		***0.025***		
**b**_before_	29.034	0.884	30.354	0.485	***0.001***	30.264	0.839	30.103	0.547	*0.380*
**b**_after_	35.504	1.462	38.854	1.166	***0.001***	29.449	0.505	28.084	0.398	***0.001***
**p value	***0.001***		***0.001***			***0.001***		***0.001***		
**ΔE_ab_**	7.404	1.519	9.708	1.192	***0.001***	1.390	0.585	2.523	0.846	***0.001***
**ΔE_00_**	7.502	1.597	19.501	1.327	***0.001***	1.317	0.511	2.454	0.632	***0.001***

Abbreviation: SD, standard deviation.

*The significance level of the difference between materials in the same period and the same process (independent t‐test) **→**.

**The significance levels of the “pre‐post measurement differences” according to the paired *t*‐test ↓.

The distribution and comparison of roughness measurements based on cigarette exposure and material type are presented in Table 3. Following conventional cigarette exposure, the surface roughness values of all specimens showed a significant increase (*p* < 0.05). After exposure to electronic cigarettes, the Ra value of the porcelain specimens decreased significantly (*p* < 0.05). While the surface roughness of the indirect composite resins increased after electronic cigarette exposure, the changes were not statistically significant (*p* > 0.05).

**TABLE 3:** The descriptive and comparative statistics of the surface roughness values.

Conventional cigarette						Electronic cigarette				
	Gingiva‐colored porcelain		Gingiva‐colored indirect composite resin			Gingiva‐colored porcelain		Gingiva‐colored indirect composite resin		
	Mean	SD	Mean	SD	*p value	Mean	SD	Mean	SD	*p value
**Ra**_before_	0.115	0.045	0.041	0.030	***0.001***	0.126	0.050	0.023	0.017	***0.001***
**Ra**_after_	0.295	0.077	0.242	0.047	***0.001***	0.102	0.035	0.036	0.032	***0.001***
**p value	***0.001***		***0.001***			***0.025***		*0.065*		
**Sa**_before_	0.668	0.288	0.304	0.333	***0.001***	0.746	0.469	0.187	0.146	***0.001***
**Sa**_after_	0.816	0.296	0.490	0.137	***0.001***	0.555	0.210	0.255	0.232	***0.001***
**p value	***0.020***		***0.008***			*0.059*		*0.189*		

Abbreviation: SD, standard deviation.

* The significance level of the difference between materials in the same period and the same process (independent *t*‐test) **→**.

** The significance levels of the “pre‐post measurement differences” according to the paired *t*‐test ↓.

**FIGURE 4:**
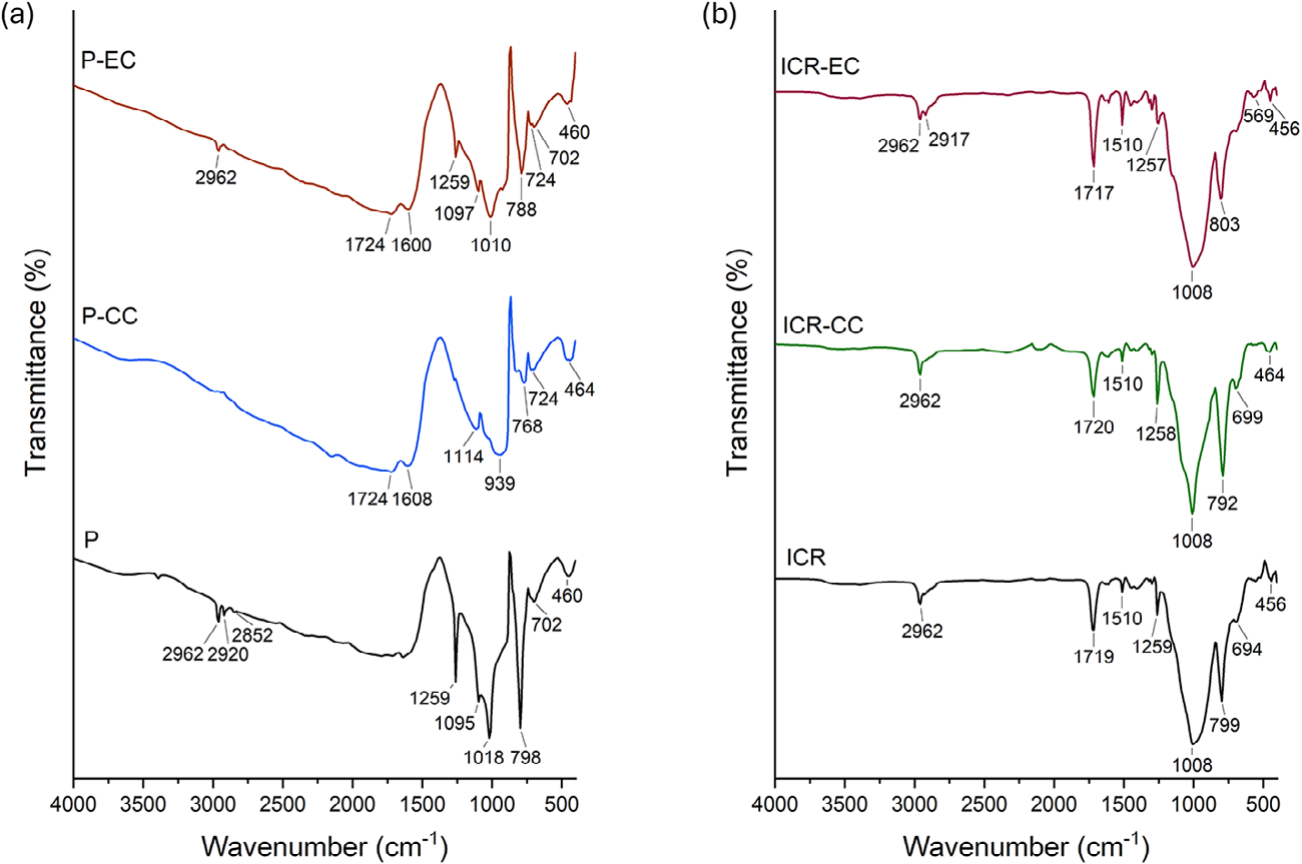
FTIR spectra of the gingiva‐colored specimens before and after exposure to conventional cigarettes (CC) and electronic cigarettes (EC): (a) Porcelain (P) specimens, (b) Indirect Composite Resin (ICR) specimens.

The FTIR spectra of the gingiva‐colored porcelain and indirect composite specimens obtained before and after exposure to conventional and electronic cigarettes are shown in Figure 4. In Figure 4a, the bands originating from the gingiva‐colored porcelain were observed at 1000–1300 cm^−1^ (asymmetric stretching mode of Si–O–Si), 770–800 cm^−1^ (symmetric stretching vibration of Si–O–Si), and 450–460 cm^−1^ (rocking vibration of Si–O–Si).^32^ After exposure to conventional cigarette smoke (CC) and electronic cigarette smoke (EC), changes were observed in the spectra. The bands around 1720 cm^−1^ and 1600 cm^−1^, associated with the stretching of C═O in carboxylic acids and C═C stretching in aromatics, were recorded for both cigarette types.^33^ Additionally, the sharp peak at 798 cm^−1^, attributed to the symmetric Si–O–Si stretching vibration, decreased in intensity after exposure, particularly to the conventional cigarette. A band around 720 cm^−1^, attributed to the presence of the leucite phase in the porcelain composition,^34^ became visible after exposure to either cigarette.

In Figure 4b, the FTIR spectrum of the indirect composite resins shows a strong band at 1008 cm⁻¹, which can be assigned to Si–O stretching vibrations, possibly due to the silicate components in the resin matrix. Additionally, the band at 1510 cm⁻¹ corresponds to the N–H deformation stretching of urethane dimethacrylate (UDMA), which remains consistent before and after exposure to both conventional and electronic cigarettes.^35^ The band at 1719 cm^−1^, assigned to free carbonyl stretching, was observed in the unexposed samples and after both cigarette exposures.

## DISCUSSION

The present study evaluated the effect of conventional and electronic cigarettes on the discoloration and surface roughness of two different gingiva‐colored materials. Significant differences in terms of discoloration and surface roughness were found among tested gingiva‐colored materials after both cigarette exposures (*p* < 0.05). Significant differences in ΔE*_ab_ and ΔE_00_ values were found among groups after conventional and electronic cigarette exposure. In addition, conventional cigarette exposure significantly increased the surface roughness of both porcelain and indirect composite resin materials (*p* < 0.05). Therefore, the null hypotheses were rejected.

The discoloration and surface roughness of dental materials can be explained by different mechanisms such as chemical and physical interactions.^7^, ^10^, ^14^ Increased surface roughness increases plaque accumulation.^12^, ^15^ Polishing and glazing processes are applied to surfaces to obtain a smooth surface. Surface treatments like autoglaze, overglaze, or manual polishing are commonly used to reduce the surface roughness of porcelain materials.^13^, ^15^ In this study, the overglaze porcelain was applied on porcelain specimens’ surfaces. The porcelain specimens’ initial surface roughness mean values were 0.1 µm. It was stated that surface sealant agents are used to reduce surface roughness, improve wettability, and enhance resistance to discoloration of resin‐based materials.^14^, ^15^ Thus, in this in vitro study, the surface sealant agent was applied to the surface of the indirect composite resin specimens to mimic the clinical procedure. According to measurements of the initial surface roughness, mean values were 0.03 µm. Both initial measurements were below the critical surface roughness threshold.^12^

It was observed that all gingiva‐colored specimens exposed to conventional cigarettes showed statistically significant more color change compared to the specimens exposed to the electronic cigarette (*p* < 0.05). The literature varies when reporting the perceptible (PT) and acceptable (AT) thresholds for color difference (ΔE) of gingiva‐colored materials.^9^, ^36^ Paravina et al. reported the PT and AT to be ΔE*_ab_≤ 3.7 and ΔE_00_ ≤ 2.8, respectively. According to the results of this study, the color changes of all gingiva‐colored materials exposed to the conventional cigarette were clearly clinically unacceptable. This finding aligns with a prior study.^29^ In the research by Vohra et al., tooth‐colored dental porcelain and composite resin samples were exposed to conventional and electronic cigarettes. All specimens exposed to the conventional cigarette were affected more than those exposed to the electronic cigarette (*p* < 0.05).

In the literature, there are various studies investigating the effects of conventional cigarettes on dental tissues, dental porcelains, composite resin materials, and artificial teeth.^10^, ^16^, ^17^, ^30^ Most studies focus on the effects of conventional cigarettes on color changes in materials, while only a few have evaluated their impact on surface roughness.^10^, ^16^, ^17^, ^19^ Different cigarette exposure procedures have been employed in these studies, such as variations in the number of cigarettes, duration, vacuum methods and airflow rates, the size of the exposure chambers where test specimens are placed, as well as cleaning, whitening, or repolishing the specimens during or after exposure.^3^, ^10^, ^17^, ^18^, ^19^, ^21^ To ensure standardization in this study, gingiva‐colored porcelain and indirect composite resins were exposed to 20 conventional cigarettes (equivalent to one pack) according to prior studies assessing color and roughness changes in materials due to conventional cigarette exposure.^10^, ^17^, ^19^ For in vitro simulation of conventional cigarette use, the CORESTA recommended method No. 22 (2 s puff duration with a 60 s interval between puffs) was utilized.^23^ To apply this method, a vacuum device with a start‐stop feature was used instead of a continuous vacuum system. Following exposure, all specimens were rinsed with distilled water for 1 min without any supplementary procedures such as whitening, brushing, or polishing, to assess the effects of cigarette exposure.^17^, ^25^

After conventional cigarette exposure, ΔL* parameters were significantly increased and Δb* parameters were decreased for all gingiva‐colored materials (p<0.05). These findings are consistent with the literature.^17^, ^20^ Nevertheless, the Δa* parameters showed a notable decrease in comparison to tooth or tooth‐colored materials. Unlike tooth‐colored materials, the decrease in the redness value (+a*) of gingiva‐colored materials can be explained by the degradation of oxide bonds in the metal oxide content of pigments due to heat and chemical reactions.^5^, ^11^

Limited studies in the literature^25^, ^29^, ^30^ evaluated the effects of electronic cigarettes on dental tissue and dental materials. However, there is no standard protocol such as exposure time, liquid type, puff counts, or puff duration for electronic cigarettes.^21^ In this study to ensure standardization of conventional and electronic cigarette exposure, a protocol with 200 puffs was applied for electronic cigarette exposure, equivalent to 20 conventional cigarettes. The CORESTA recommended method No. 81 was followed, with a puff duration of 3 s and a 30 s interval between puffs.^22^ Electronic cigarettes work based on the principle of heating a liquid containing water, glycerin, vegetable glycerol, nicotine, and various flavors, which is then aerosolized using an atomizer.^2^ The oxidation of flavors and nicotine in the liquid changes the color. Pintado‐Palomino et al. reported an increase in yellowness (+b*) values when using tobacco‐flavored liquids, and an increase in blueness (‐b*) values when using menthol‐flavored liquids.^25^ In this study, similar to the research of Pintado‐Palomino et al., the menthol‐flavored liquid was used, and after the exposure, a statistically significant increase in the blueness (‐b*) value of gingiva‐colored materials was observed. After electronic cigarette exposure, a statistically significant increase in the “L” and “a” parameters of gingiva‐colored porcelain and indirect composite resin samples was observed (*p* < 0.05). In the literature, different results have been reported regarding the change in the “L” parameter of tooth‐colored materials after exposure to electronic cigarettes, with Vohra et al. reporting a statistically significant decrease, while Alrabeah et al. reported an increase in “L” value.^26^, ^29^ The reason for this discrepancy may be attributed to a lack of knowledge regarding the chemical composition of the liquids that may be generated during the aerosolization process.^2^ Therefore, electronic cigarette liquids with different compositions may have different effects on the discoloration and surface properties of dental tissues and materials. Similarly, while the a* parameters decrease in dental tissues and tooth‐colored materials, the statistically significant increase observed in this study for gingiva‐colored materials could be explained by differences in the liquid content.

After the conventional cigarette exposure, the surface roughness values of all gingiva‐colored materials were significantly increased (*p* < 0.05). These results were in line with prior studies.^17^, ^19^ The increase in surface roughness can be explained by the fact that conventional cigarettes cause micro cracks and porosities on the surfaces, which retain debris in surface irregularities.^17^

In this study, a decrease in the surface roughness of gingiva‐colored porcelain resins after electronic cigarette exposure was observed (*p* < 0.05). Electronic cigarette liquids contain high levels of glycerol and propylene glycol.^2^ It was stated that an increase in temperature on rough ceramic surfaces increased the mobility of glycerol droplets. It has been reported that this decreased the contact angle of glycerol with the surface and increased its wettability.^37^ In this study, the decrease in surface roughness values of gingiva‐colored porcelains can be explained as the results of glycerol obtained by aerosolizing the electronic cigarette liquid covering the rough porcelain surface and thus camouflaging the roughness on the porcelain surface.

To the best of our knowledge, this is the first study that evaluated the effects of electronic cigarettes on the surface roughness of any dental materials. There are various studies in the literature that exhibit that different nicotine ratios cause varying effects on color change parameters.^25^ The aerosol of electronic cigarettes contains reactive oxygen species, such as free radicals and peroxides, which are formed as a result of the thermal degradation of the e‐liquid.^2^ The aerosol of electronic cigarettes may have caused some degradation on the Optiglaze surface sealant agent containing methylmethacrylate. The increase in surface roughness parameters of gingiva‐colored indirect composite resins can be explained by this mechanism to the results of this study. However, this increase is not statistically significant (*p* > 0.05). Prolonging the exposure duration could affect the results differently.

The changes observed in the FTIR spectra after cigarette exposure suggest alterations in the surface chemical structure of both types of specimens. For instance, the bands corresponding to organic compounds on porcelain, such as C═O and C═C stretching, indicate that cigarette smoke contributes to the adsorption of organic molecules onto the surface. This is also supported by the decrease in intensity of the symmetric Si–O–Si stretching peak at 798 cm⁻¹ in the porcelain specimens, particularly after exposure to conventional cigarette smoke. Although the functional groups in the indirect composite resins exhibited relatively smaller changes, some shifts in peak intensities were noted in Figure 4b.

This study is the first to investigate the effects of conventional and electronic cigarettes on gingiva‐colored porcelain and indirect composite resins in the literature. In vitro conditions were used to simulate the smoking process in this study. The limitations include the lack of exposure to oral fluids and the absence of exposure to temperature or pH changes that could occur in the oral environment, such as eating, drinking, or brushing teeth. Additionally, to ensure standardization of the experiment, the exposure time was limited to the equivalent of one pack of conventional cigarettes and one pack of electronic cigarettes, similar to the literature. Different results may be obtained by conducting the study in vitro and in vivo with additional steps or by extending the exposure durations. Differences in color and surface parameters can be objectively evaluated by exposing both dental and gingiva‐colored materials to conventional and electronic cigarettes under the same conditions. Advanced studies are planned to assess the effects under conditions that simulate the oral environment.

## CONCLUSIONS

Conventional cigarette exposure leads to substantial color change and increased surface roughness, highlighting the need for caution in patients with these prosthetic materials. Gingiva‐colored porcelain specimens exhibited better resistance to color and surface roughness changes compared to indirect composite resin, especially when exposed to electronic cigarette smoke. Electronic cigarettes cause significantly less color and surface roughness changes in both materials compared to conventional cigarettes. Clinicians should consider the patient's smoking habits when selecting gingiva‐colored materials, as material performance varies significantly depending on exposure type.

## CONFLICT OF INTEREST STATEMENT

The authors declared no conflict of interest.
